# Body weight, frailty, and chronic pain in older adults: a cross-sectional study

**DOI:** 10.1186/s12877-019-1149-4

**Published:** 2019-05-24

**Authors:** Cheng Chen, Almut G. Winterstein, Roger B. Fillingim, Yu-Jung Wei

**Affiliations:** 1 Department of Pharmaceutical Outcomes and Policy, College of Pharmacy, University of Florida, Gainesville, USA; 2 Center for Drug Evaluation and Safety, College of Pharmacy, University of Florida, Gainesville, USA; 3 Department of Epidemiology, College of Public Health and Health Professions & College of Medicine, University of Florida, Gainesville, USA; 4 Department of Community Dentistry and Behavioral Science, College of Dentistry, University of Florida, Gainesville, USA

**Keywords:** Unhealthy weight, BMI, Pain, Frailty, Older adults

## Abstract

**Background:**

There exists limited data on the association between unhealthy body weight and chronic pain, and whether this association is explained by frailty status of older adults.

**Methods:**

We included older adults aged ≥65 years from the 1999–2004 National Health and Nutrition Examination Survey (NHANES). Chronic pain was defined by self-reported pain lasting for ≥3 months in the past year. Body mass index (BMI) was categorized as underweight, normal, overweight, and obese. Participants were dichotomized as frail or non-frail based on a validated frailty index calculated as the proportion of the number of deficits present to a total of 45 possible deficits ascertained in NHANES. We used modified Poisson regression models to estimate prevalence ratios (PRs) and their 95% confidence intervals (CIs).

**Results:**

Of 3693 older participants, one in six (15.9%) experienced chronic pain, with higher prevalence among the underweight (24.6%) and obese (20.2%) group. Frailty versus non-frailty was independently associated with BMI (PR = 1.25, 95% CI = 1.16–1.36 for underweight; and PR = 1.15, 95% CI = 1.07–1.22 for obese), and chronic pain (PR = 2.84, 95% CI = 2.18–3.69). After adjustment for frailty, the association between BMI and chronic pain decreased from PR = 1.82 to 1.64 for the underweight and 1.41 to 1.33 for the obese group. We did not observe an interaction effect between frailty and BMI.

**Conclusions:**

Unhealthy body weight was associated with increased chronic pain and the associations were partially explained by frailty status of older adults. Our findings generate hypotheses for further investigations of the interplay of these chronic conditions in older adults.

**Electronic supplementary material:**

The online version of this article (10.1186/s12877-019-1149-4) contains supplementary material, which is available to authorized users.

## Background

Chronic pain is one of the most common conditions experienced by older adults. More than 50% of community-dwelling older adults and 80% of nursing home residents report pain regularly [1, 2]. Unlike younger adults, older adults tend to experience pain at multiple sites and have more than one pain causes [3]. Common causes of chronic geriatric pain include musculoskeletal disorders (such as osteoarthritis and rheumatoid arthritis), diabetic neuropathies and postherpetic neuralgia, cancer and cancer treatment, and advanced stages of chronic conditions that induce pain, such as congestive heart failure and end-stage renal disease [4]. Older adults who experienced acute pain from surgery, trauma, or injury may also develop chronic pain if the pain signals remain active for more than 3 months [5].

The diversity of chronic pain conditions encountered by older adults could have different effects on their body weights. Cancer-related chronic inflammation likely leads to chronic pain and underweight [6], and diabetes-associated obesity increases the burden of weight-bearing and worsens the chronic pain of lower back and lower limbs [7]. While excessive body weight is suggested as a risk factor for chronic pain among adults [8, 9], there is limited data to understand such an association among the United States (US) geriatric population. A previous small-scale study of 840 US older adults aged over 70 observed that obese persons had two-to-four-fold greater odds of chronic pain than their normal-weight peers [10]. Besides, we know very little about the relationship between chronic pain and being underweight—the other end of the body weight spectrum, which is particularly common among frail elderly. Understanding this relationship is essential because one-in-sixty US older adults are underweight due to malnutrition and morbidity [11]. In anticipation of a growing older population with unhealthy weight as baby boomers age, delineating the relationship between unhealthy weight and chronic pain may inform further research and interventions on pain management in the geriatric population.

Frailty was proposed as a clinically recognizable state of increased vulnerability resulting from age-associated decline in functional, cognitive, and physiologic reserves. It has been operationalized as a frailty index by counting the number of deficits accumulated to quantify the degree of susceptibility for adverse health outcomes such as death [12]. Based on a frailty index measure, one in three US older adults is considered to be frail [13]. Frailty index, body weight, and pain appear to be intertwined. Excessive low or high body weight could lead to muscle weakness and reduced physical activities, which predisposes older adults to increased risk for frailty [14]. The state of frailty is also a risk factor for chronic pain [15–17]. Yet the role of frailty in explaining the association between body weight and chronic pain remains unexplored.

This study aimed to investigate the association between unhealthy weight and chronic pain in a representative sample of US older adults. We also attempted to evaluate whether frailty explains or modifies this association.

## Methods

### Study design and data

This cross-sectional study used National Health and Nutrition Examination Survey (NHANES) data from three survey cycles: 1999–2000, 2001–2002, and 2003–2004. We use these survey cycles because they collected data on our key variables—chronic pain and body weights/heights, as well as information on laboratory results, physical and mental comorbidities to allow the construction of a frailty index. NHANES applies a multistage, probability cluster design to obtain a representative sample of the noninstitutionalized US population. The National Center for Health Statistics Ethics Review Board approved the NHANES study and documented consent was obtained from participants. Data is made available publicly for research purposes in a de-identified fashion.

### Sample selection

The study sample included participants 1) whose reported age was 65 or older in any of the three NHANES survey cycles, 2) who had complete information on our key variables—body mass index (BMI) and chronic pain, and 3) who had non-zero sample weights to extrapolate nationally-representative estimates [18]. To measure the frailty index, a potential intermediator or modifier of the association between BMI and chronic pain, we further included eligible sample who had complete, non-missing information on 80% or more of the 45 conditions and symptoms that were collected at NHANES and used for the frailty index measure (see details in *Frailty Index Measure*).

### Measures

#### Body mass index

The independent variable of interest was BMI. Individuals who participated in NHANES had their weight and height measured by trained staff at designated mobile examination centers. Individual BMI was calculated from measured body weight and height as weight (in kg) divided by height (in meters) squared. Following the Centers for Disease and Control and Prevention (CDC) classification, we categorized BMI into four groups: underweight (BMI <  18.50), normal (18.50 ≤ BMI < 25; the reference group), overweight (25 ≤ BMI < 30), and obese (BMI ≥30).

#### Chronic pain

The dependent variable of interest was chronic pain, defined as pain lasting for more than 3 months based on self-report data that were only collected in 1999–2004 NHANES. Adult participants were interviewed about whether they had pain conditions and how long the pain persisted in the past year. For those who reported any existence of pain, we further reported their location and duration of pain. Pain duration in NHANES was measured by the question: “How long experience this pain?” The answer was coded as “no pain/less than 24 hours”, “more than 24 hours but less than a month”, “at least 1 month but less than 3 months”, and “greater than 1 year”. We classified participants who reported free of chronic pain but had a lifetime history of using pain reliver products for more than 1 year as having no current chronic pain [19]. The lifetime history of chronic pain medication use collected in NHANES 1999–2004 might have happened early in life or later when the interviews were conducted. Lack of documentation on specific time period for chronic analgesic use prevented from determining whether participants were having chronic pain during our study period.

#### Frailty index (FI) measure

We adopted an FI measure previously constructed using NHANES [13, 20]. The FI was calculated as a proportion of the number of deficits present in an individual to a total of 46 possible health deficits considered. These health deficits, pre-selected based on a standard protocol, include comorbidities, reduced physical function, abnormal laboratory results, and use of multiple prescription drugs. The NHANES–based FI has been validated against adverse health outcomes including disability, self-reported health and healthcare utilization [13].

In our FI measure, we excluded one item– “difficulty pushing or pulling large objects” because this variable was not collected in NHANES 1999–2004, leaving a total of 45 items in the denominator. Following the previous NHANES-version FI, we also excluded study participants who had a missing value of ≥20% (i.e., nine variables) of the 45 frailty items. Our FI ranged between zero (total fitness) and one (total frailty). In the analysis, we dichotomized older adults as frail versus non-frail using a validated cut-off of 0.21, which optimally discriminates between frail and non-frail individuals [13, 21].

#### Covariates

We considered covariates potentially associated with BMI and pain, including sociodemographics, smoking status, alcohol consumption, cancer (yes versus no), and disease burden. Sociodemographic characteristics included age (65–69, 70–79, or ≥ 80 years), gender (male or female), race/ethnicity (non-Hispanic Whites, non-Hispanic Blacks, Mexican American, other Hispanic, or other), education level (less than high school, high school, some college, or college graduate), and family income-to-poverty threshold ratio (< 1, 1 to 2, > 2 to < 4, and ≥ 4, with a smaller number indicating lower income status). Smoking status was classified as never smokers (smoked < 100 cigarettes in their lifetime and not currently smoking), current smokers (smoked ≥100 cigarettes in their lifetime and currently smoking), and former smokers (smoked ≥100 cigarettes in their lifetime but not currently smoking) [22]. Alcohol consumption was classified according to the average number of alcoholic drinks/day as no use (zero drinks/day), moderate users (≤one drink/day for females; ≤ two drinks/day for males), and heavy users (>one drink/day for females, >two drinks/day for males) [23]. Disease burden was measured as the total number of 12 chronic conditions that were commonly seen in older adults and collected during the three NHANES survey cycles, including arthritis, osteoporosis, hypertension, diabetes, coronary heart disease, heart attack, thyroid condition, stroke, angina, depression, heart failure, and kidney disease. Due to the skewed distribution of the number of chronic diseases, we categorized the number of chronic conditions into four mutually exclusive groups (0, 1, 2, and ≥ 3) and included it as a categorical variable in the models.

### Statistical analysis

In the overall sample and within each BMI group, we described individual characteristics and frailty level. We also reported the prevalence of chronic pain, specific pain sites, duration of pain, and the average number of pain sites. Differences in the variables of interest among BMI groups were examined using Chi-squared tests for categorical variables and ANOVA for continuous variables. To provide national estimates, we incorporated survey weights in the descriptive analyses.

Before statistical analyses, we imputed missing values on covariates (i.e., education, alcohol consumption, smoking, and cancer) using multiple imputations. Since we had a substantial proportion (33.9%) of the study sample with missing data on alcohol consumption, to increase the efficiency of point estimators, we imputed a maximum number of 25 replicates as defaulted by statistical software.

To understand the relationships among BMI (independent variable), chronic pain (dependent variable) and frailty (potential intermediator or modifier), we constructed three separate modified Poisson models with robust error variance and reported adjusted prevalence ratios (PRs) with 95% confidence intervals (CIs) for association estimates. We used modified Poisson regression to calculate PRs rather than logistic regression for odds ratios (ORs) because the latter estimate tends to overestimate the effect when study sample is population-based according to statistical literature [24, 25]. PR is a more conservative, less-biased estimated than OR in studies using nationally representative, population-based sample surveys, such as NHANES.

We fitted three regression models. First, to determine the relationship between BMI and chronic pain, we regressed chronic pain on BMI controlling for covariates. Second, to determine the association between frailty and BMI, we regressed frailty on BMI with covariate adjustment. Finally, to examine whether the relationship of BMI with chronic pain was explained, in part, by frailty, we repeated the same analysis and included frailty as an explanatory variable. We calculated the percent change in the PRs between chronic pain and BMI before and after frailty adjustment. In the model with frailty adjustment, we further examined the interaction effect of BMI and frailty on the prevalence of chronic pain. We tested model fit improvement attributable to the BMI*frailty interaction using likelihood ratio tests with significance levels set at < 0.05. The associations between BMI and chronic pain were stratified by frailty levels and reported within each stratum. As a sensitivity analysis, we repeated all regression models exclusively among older adults without missing values of covariates. Also, because BMI and FI-related variables were collected in all NHANES cycles, we examined the association between BMI and frailty using the latest three survey rounds of NHANES (2011–2012, 2013–2014, 2015–2016).

With 80% power and a 5% significance level for two-sided, our study sample size (i.e., 982 obese subjects and 1029 normal weight subjects) showed a sufficient statistical power to detect a minimal effect on the PR (1.16) for obesity in relation to chronic pain. All analyses were performed using SAS version 9.4, with PROC GENMOD for modified Poisson analyses and PROC MI for the multiple imputation of missing data. *P* < .05 were considered statistically significant, and all tests were two-sided.

## Results

### Characteristics of the study sample

We identified 3693 eligible older respondents (Additional file 1). Of the older adult sample, over two-thirds (72.1%) had unhealthy weight, with the majority being overweight (38.0%) or obese (26.6%), but 7.6% were underweight (Table 1). Most characteristics differed significantly among the four BMI groups. For instance, compared to the normal, the obese group was younger, had more Blacks, with lower education and fewer people having a family income-to-poverty ratio of ≥4, but tended to be none- or former smokers, and not have cancer. Conversely, the underweight (versus the normal) were older, had more females and non-Whites, and tended to be current smokers and have cancer. The proportions of individuals with an education level lower than high school and a family income-to-poverty ratio of ≤1 were higher among the underweight (47.3 and 29.6%) than the normal weight (29.8 and 23.1%). The proportion of older adults who had at least three chronic conditions was 42.6% for the underweight, 43.6% for the obese, and 30.1% for the normal weight.

**Table 1:** Characteristics of older respondents in 1999–2004 NHANES

Characteristics	Total Sample (%)	BMI Groups				*P*-Value
		Normal (%)	Underweight (%)	Overweight (%)	Obese (%)	
	*N* = 3693	*n* = 1029	*n* = 280	*n* = 1402	*n* = 982	
Age group, y						<.0001
65–69	29.1	23.8	9.6	29.6	39.0	
70–79	47.0	44.3	45.7	48.5	47.9	
≥ 80	23.9	31.8	44.7	22.0	13.1	
Male sex	42.7	38.8	36.0	49.2	39.5	<.0001
Race/Ethnicity						0.002
Non-Hispanic White	82.8	83.5	77.1	84.0	81.9	
Non-Hispanic Black	7.7	5.6	9.4	6.8	10.5	
Mexican American	2.8	2.7	2.8	2.8	2.8	
Other Hispanic	4.1	3.3	8.6	4.6	3.1	
Others^a^	2.7	5.0	2.1	1.8	1.6	
Education^b^						0.008
< High school	32.0	29.8	47.3	30.3	32.8	
High school	29.3	29.8	24.5	28.4	31.1	
Some college	22.2	23.5	18.9	22.6	21.4	
College	16.5	16.9	9.3	18.8	14.7	
Family income-to-poverty ratio						0.001
< 1	21.2	23.1	29.6	17.9	21.8	
1 - ≤2	28.4	26.6	39.6	27.1	28.9	
> 2 - < 4	29.6	28.1	20.3	32.1	30.2	
≥ 4	20.8	22.1	10.5	22.9	19.1	
Alcohol use^b^						0.001
No use	20.5	20.6	22.6	20.5	19.8	
Moderate use	35.9	38.2	23.6	37.7	34.4	
Heavy use	9.7	10.3	7.2	11.0	8.1	
Smoking^b^						0.002
Never smoker	49.1	49.9	45.6	48.2	50.3	
Former smoker	41.8	38.5	39.0	43.7	43.0	
Current smoker	9.0	11.4	15.1	7.8	6.6	
Cancer (Yes)^b^	23.6	24.0	27.0	23.7	22.2	0.082
Number of chronic conditions^c^						<.0001
0	14.4	18.8	13.5	14.4	10.3	
1	25.3	28.1	16.8	29.0	19.8	
2	24.9	22.9	27.1	24.9	26.2	
≥3	35.4	30.1	42.6	31.8	43.6	
Frailty (Yes)	57.2	52.1	80.2	51.4	64.4	<.0001
Chronic pain	15.9	11.7	24.6	14.2	20.2	<.0001
Pain by body location						
Joint	54.7	47.8	55.0	53.7	63.1	<.0001
Back	13.7	10.5	16.7	12.3	17.8	0.002
Legs/feet	10.4	7.5	12.7	10.3	12.7	0.010
Arms/hands	3.2	1.9	5.5	3.0	4.1	0.057
Headache	2.3	1.8	3.1	2.2	2.6	0.732
Duration						<.0001
No pain/Pain less than 24 h	79.7	82.2	76.1	81.5	75.7	
<1 month	3.7	4.7	1.3	4.1	2.6	
1–3 months	1.9	1.5	1.0	1.3	3.5	
3 months-1 year	3.2	2.3	1.1	3.2	4.8	
> = 1 year	11.5	9.4	20.5	9.8	13.5	
Average number of pain sites per person						<.0001
Mean (Standard Deviation)	0.88(0.02)	0.73(0.03)	1.01(0.09)	0.85(0.04)	1.05(0.04)	

*Abbreviations.* BMI=Body mass index

^a^Others included Asian, the natives of North American, multi-racial, and individuals with other or unknown races and ethnicities

^b^Imputed those with missing values for regression analysis

^c^Chronic conditions of interest include arthritis, osteoporosis, hypertension, diabetes, coronary heart disease, heart attack, thyroid condition, stroke, angina, depression, heart failure, and kidney disease

### Prevalence of chronic pain/pain sites and its association with BMI

Table 1 also shows the prevalence of chronic pain, pain sites, and duration of pain by BMI groups among older adults. The overall prevalence of chronic pain was 15.9%, with the highest estimate observed in underweight older adults (24.6%), followed by obese (20.2%) and overweight (14.2%) persons. Compared to the normal weight older adults, those with unhealthy weight were more likely to report pains at joints, backs, and legs/feet, as well as having a higher average number of pain sites. The proportion of individuals who experienced pain for 1 year or longer was 22.4% for the underweight, 15.1% for the obese, 11.1% for the overweight, and 9.4% for the normal weight group. Multivariable results indicated both underweight and obese older adults (versus normal weight) had a higher probability of experiencing chronic pain (PR = 1.64, 95% CI = 1.24–2.17; PR = 1.33, 95% CI = 1.06–1.66, respectively) (Table 2).

**Table 2:** Multivariable analysis of the association between BMI and frailty among US older adults (N = 3693)

Risk Factors	Frailty vs. Non-frailty	
	Adjusted PR	95% CI
BMI		
Normal	1.00	–
Underweight	1.25	1.16–1.36
Overweight	0.97	0.91–1.03
Obese	1.15	1.07–1.22
Age group, y		
65–69	1.00	–
70–79	1.22	1.13–1.31
≥ 80	1.55	1.44–1.67
Gender		
Male	1.00	–
Female	1.00	0.95–1.05
Race/Ethnicity		
Non-Hispanic White	1.00	–
Non-Hispanic Black	0.92	0.86–1.00
Mexican American	1.02	0.95–1.10
Other Hispanic	0.93	0.79–1.10
Others^a^	1.16	1.00–1.34
Education^b^		
< High school	1.00	–
High school	0.94	0.88–1.00
Some college	0.94	0.88–1.00
College	0.96	0.88–1.04
Family income-to-poverty ratio		
< 1	1.00	–
1–2	0.96	0.91–1.02
> 2 - < 4	0.89	0.82–0.96
≥ 4	0.96	0.88–1.04
Alcohol use^b^		
Nonuse	1.00	–
Moderate use	0.93	0.87–1.00
Heavy use	0.94	0.84–1.04
Smoking^b^		
Never smoker	1.00	–
Former smoker	1.07	1.01–1.12
Current smoker	1.14	1.03–1.25
Cancer^b^		
No	1.00	–
Yes	1.20	1.14–1.27
Number of chronic conditions^c^		
0	1.00	–
1	1.93	1.60–2.32
2	3.17	2.66–3.78
≥3	4.45	3.75–5.28

*Abbreviations. BMI* Body mass index, *PR* Prevalence ratio, *CI* Confidence interval

^a^Others included Asian, the natives of North American, multi-racial, and individuals with other or unknown races and ethnicities

^b^Imputed those with missing values for regression analysis

^c^Chronic conditions of interest include arthritis, osteoporosis, hypertension, diabetes, coronary heart disease, heart attack, thyroid condition, stroke, angina, depression, heart failure, and kidney disease

### Frailty and its association with BMI

Of the older adult sample, over half (57.2%) were frail (Table 1). We observed a significantly higher proportion of frail patients among the underweight (80.2%) and obese group (64.4%), as compared with that of normal weight group (52.1%). In multivariable regression, we observed underweight and obese older adults had a higher probability of frailty (PR = 1.25, 95% CI = 1.16–1.36; PR = 1.15, 95% CI = 1.07–1.22, respectively) (Table 2). A similar finding was observed in more recent three survey rounds of NHANES (2011–2012, 2013–2014, 2015–2016) (Additional file 2) as well as our sensitivity analysis where only complete cases without missing values were analyzed *(*Additional file 3*).*

### BMI and chronic pain by frailty

When we included both BMI and frailty in the multivariable model, frailty was significantly associated with a nearly 3-fold greater probability of chronic pain (PR = 2.84, 95% CI = 2.18–3.69) (Table 3). After adjustment for frailty, the PRs of the associations between chronic pain with being underweight or obese were reduced from 1.82 (95% CI = 1.37–2.42) to 1.64 (95% CI = 1.24–2.17) and 1.41 (95% CI = 1.13–1.77) to 1.33 (95% CI = 1.06–1.66), respectively. The relative percent change in the PRs of chronic pain and BMI before and after the frailty adjustment was 10.1% for the underweight, and 6.0% for the obese group.

**Table 3:** Multivariable analysis of the association between BMI and chronic pain, with and without adjustment for frailty (*N* = 3693)

Risk Factors	Chronic Pain vs No Chronic Pain			
	Model 1 without Frailty		Model 2 with Frailty	
	PR	95% CI	PR	95% CI
Frailty				
No	–		1.00	–
Yes			2.84	2.18–3.69
BMI				
Normal	1.00	–	1.00	–
Underweight	1.82	1.37–2.42	1.64	1.24–2.17
Overweight	1.13	0.90–1.41	1.14	0.92–1.43
Obese	1.41	1.13–1.17	1.33	1.06–1.66
Age group, y				
65–69	1.00	–	1.00	–
70–79	0.89	0.73–1.07	0.82	0.68–0.99
≥ 80	0.77	0.61–0.96	0.63	0.51–0.80
Gender				
Male	1.00	–	1.00	–
Female	1.11	0.93–1.32	1.12	0.94–1.32
Race/Ethnicity				
Non-Hispanic White	1.00	–	1.00	–
Non-Hispanic Black	0.83	0.65–1.05	0.86	0.68–1.08
Mexican American	0.67	0.51–0.88	0.67	0.51–0.88
Other Hispanic	0.91	0.57–1.47	0.97	0.61–1.56
Others^a^	1.26	0.76–2.08	1.15	0.69–1.91
Education^b^				
< High school	1.00	–	1.00	–
High school	0.96	0.77–1.19	1.0	0.80–1.24
Some college	1.00	0.80–1.26	1.04	0.83–1.30
College	1.04	0.78–1.39	1.07	0.81–1.42
Family income-to-poverty ratio				
< 1	1.00	–	1.00	–
1–2	0.86	0.70–1.06	0.88	0.72–1.08
> 2 - < 4	0.86	0.69–1.07	0.89	0.72–1.11
≥ 4	0.81	0.62–1.06	0.85	0.65–1.12
Alcohol use^b^				
No use	1.00	–	1.00	–
Moderate use	1.10	0.85–1.42	1.14	0.89–1.46
Heavy use	1.17	0.80–1.72	1.21	0.83–1.77
Smoking^b^				
Never smoker	1.00	–	1.00	–
Former smoker	1.05	0.87–1.26	1.03	0.86–1.24
Current smoker	1.08	0.80–1.46	1.03	0.76–1.38
Cancer^b^				
No	1.00	–	1.00	–
Yes	1.068	0.87–1.28	0.97	0.80–1.18
Number of chronic conditions^c^				
0	1.00	–	1.00	–
1	2.47	1.51–4.02	2.02	1.24–3.30
2	3.90	2.43–6.25	2.50	1.53–4.07
≥3	6.45	4.09–10.18	3.39	2.09–5.48

*Abbreviations. BMI* Body mass index, *PR* Prevalence ratio, *CI* Confidence interval

^a^Others included Asian, the natives of North American, multi-racial, and individuals with other or unknown races and ethnicities

^b^Imputed those with missing values for regression analysis

^c^Chronic conditions of interest include arthritis, osteoporosis, hypertension, diabetes, coronary heart disease, heart attack, thyroid condition, stroke, angina, depression, heart failure, and kidney disease

Stratifying by frailty level, we observed significant associations between chronic pain and BMI exclusively in the frail older adults (PR = 1.68, 95% CI = 1.25–2.26 for underweight; PR = 1.33, 95% CI = 1.04–1.69 for obese). We did not observe an interaction effect between frailty and BMI on chronic pain (overall *P*-value = 1.00) (Table 4). Similar findings were shown in the sensitivity analysis (Additional files 4 and 5).

**Table 4:** Adjusted^a^ associations between BMI and chronic pain stratified by frailty level among US older adults

	Chronic Pain vs No Chronic Pain					
BMI	Overall (N = 3693)		Frail (*n* = 2114)		Non-Frail (*n* = 1579)	
	PR	95% CI	PR	95% CI	PR	95% CI
Normal	1.00	–	1.00	–	1.00	–
Underweight	1.64	1.24–2.17	1.68	1.25–2.26	1.35	0.42–4.32
Overweight	1.14	0.92–1.43	1.19	0.94–1.52	0.97	0.56–1.66
Obese	1.33	1.06–1.66	1.33	1.04–1.69	1.47	0.82–2.62
	Overall P-value for the interaction term (BMI* frailty) =1.000					

*Abbreviations. BMI* Body mass index, *PR* Prevalence ratio, *CI* Confidence interval

^a^Adjusted for age, gender, race/ethnicity, education level, family income-to-poverty ratio, alcohol use, smoking, cancer, and number of chronic conditions

## Discussion

Using a nationally representative sample from NHANES, we assessed the association between unhealthy body weight and chronic pain among US older adults. We found that compared to those with normal weight, obese older adults were 33% more likely to report chronic pain, which is consistent with previous cross-sectional studies [10, 26]; and that underweight older adults were 64% more likely to report chronic pain, suggesting the underweight may carry the same risks as obese older adults.

In older adults, the presentation of chronic pain seems different between the underweight and the obese group. Chronic pain tended to be localized in certain weight-bearing body parts (i.e., back and lower extremities including legs, ankles, and feet) of obese older adults. Localized back pain among obese older adults is commonly due to degeneration of joints in the spine [27], and localized lower extremity pain can be caused by different injuries and illness, including spinal conditions [28], peripheral arterial disease [29], and diabetic neuropathic conditions [30]. Increased pressure of extra weight on the back and lower extremities could further exacerbate and perpetuate chronic pain among older adults [31].

On the other hand, chronic pain tended to be generalized throughout the body parts of underweight older adults, including upper parts (e.g., head, arms/hand, and abdomen), joint/back, and lower extremities. The underlying reasons for the generalized pain in the underweight group remain elusive but may be attributable to pain-related comorbidities such as osteoporosis and cancer, which are prevailing among older adults [32, 33].Underweight is a risk factor for osteoporosis in the older population [34–36], which can induce weak muscle strength and create tension in the muscular and skeletal structures, predisposing individuals to bone and muscle pain all over the body [37]. For patients with malignant cancers that require chemotherapy, a substantial weight reduction often occurs, and the development of chronic pain is likely after cancer treatment [6]. This clinical feature was supported by our finding that a higher proportion of older adults with cancer was observed in the underweight group compared with other BMI groups. After adjusting for cancer, we still observed a positive association between underweight and chronic pain. Our finding highlights the importance of pain management in the underweight elderly, a subpopulation that is growing as people age, yet has been understudied.

Our study reported that over half (57.2%) of participants aged ≥65 years were categorized as being frail based on a validated NHANES-version FI algorithm. The frailty estimate fell into range (4.2–59.1%) observed in the literature using FI algorithm [38]. When examining the relationship between frailty and body weight, we found a U-shaped curve, with higher frailty observed among underweight and obese older adults, a finding in parallel with results in previous studies [39, 40]. In our sample, frailty was also identified as a strong, independent risk factor for chronic pain, which echoes earlier findings [15–17, 41].

We further investigated the role of frailty in the association between body weight and chronic pain among older adults. Adding FI to the models decreased associations by 6 to 10%, suggesting frailty index may confound the association between body weight and chronic pain. When stratifying by frailty level, we observed that the BMI-pain association emerged exclusively among the frail respondents, although the 95% CIs were wide. Literature has indicated that frail older adults are systematically different from non-frail ones regarding their response to an outside stressor, with the former manifesting increased vulnerability, dependency, and mortality [42]. In the analysis of the interaction between frailty and BMI on chronic pain, the overall *p*-value for the interaction term was non-significant in part due to the insufficient sample size of the non-frail underweight older adults. Studies using a larger sample size are needed to test whether frailty modifies the association between BMI and pain.

Our study contributes to the existing literature by exploring the interplay of body weight, chronic pain, and frailty. In geriatric care, physicians may treat underlying causes and risk factors while managing chronic pain. Clinical trials have shown that treating contributing factors such as depression decreases pain while improving functional status and quality of life [43]. Considering the high prevalence and the significant impact associated with chronic pain, understanding its risk factors is essential. Our study results indicate that excessive weight gain and loss are risk factors for chronic pain, and frailty may further magnify that pain. Different interventions for chronic pain targeted to obese and underweight older adults should be considered.

This study has several strengths. We used a nationally representative sample; therefore, our results can be generalized to the US non-institutionalized older adults. With NHANES, we were able to control for potential covariates (e.g., alcohol consumption, and smoking status) which have not been considered in prior literature to reduce the effect of confounding. Furthermore, NHANES data provide rich information on health deficits, which allowed us to measure frailty.

This study also has several limitations. First, the cross-sectional nature makes it difficult to discern the temporal relationships among BMI, chronic pain, and frailty. Future studies with longitudinal designs are warranted to examine the temporal relationships among these three conditions. Second, we operationalized unhealthy weights using BMI, a measure that may not accurately define unhealthy weight. Literature has suggested that compared to BMI, waist circumference, a proxy of visceral adipose tissues, is a better measure of obesity and more closely associated with obesity-related chronic diseases [44]. Third, variables, including chronic pain and comorbidities were self-reported and susceptible to misclassification. As with other observational studies, there is concern for unmeasured confounders such as occupation and family history. Fourth, there is a lack of acute injury information in NHANES, and possible bias may be introduced by including participants who experienced acute injury only. Finally, the data were from 1999 to 2004, which may not provide the latest estimate of prevalence. NHANES did not collect pain-related variables after 2004, which limits the generalizability of our findings to more recent years. It is noteworthy that the prevalence of obesity among older adults is increasing from 27.6% in 1999–2004 to 35.7% in 2011–2016 (Additional file 6), highlighting the need to understand and manage obesity-related chronic conditions in the aging population. Our results may serve as a preliminary for future studies using more robust methods with contemporaneous data to investigate the relationships among body weight, frailty, and chronic pain in the geriatric population.

## Conclusions

In conclusion, chronic pain was associated with both underweight and obesity in US older adults. Frailty partially explained the associations between unhealthy weight and chronic pain. Our findings warrant further analysis in longitudinal studies with large elderly samples that allow examination of the interplay among BMI, chronic pain, and frailty in the older adult population.

## Additional files

Additional file 1: Sample selection flow chart (DOCX 21 kb)

Additional file 2: Adjusted association between BMI and frailty among US older adults using NHANES 2011–2016 (DOCX 20 kb)

Additional file 3: Adjusted association between BMI and frailty among US older adults who had no missing values on covariates of interest (DOCX 13 kb)

Additional file 4: Adjusted association between BMI and chronic pain, with and without adjustment of frailty among older adults who had no missing values on covariates of interest (DOCX 15 kb)

Additional file 5: Adjusted association between BMI and chronic pain in older adults who had no missing values on covariates of interest, overall and stratified by frailty (DOCX 14 kb)

Additional file 6: Comparison of characteristics of older respondents in 1999–2004 NHANES versus in 2011–2016 NHANES (DOCX 21 kb)

## Abbreviations

BMI: Body mass index
CI: Confidence interval
FI: Frailty index
NHANES: National Health and Nutrition Examination Survey
PR: Prevalence ratios
