# Supplementary material for: Body weight, frailty, and chronic pain in older adults: a cross-sectional study
Source: BMC Geriatr. 2019 May 24;19:143. doi: 10.1186/s12877-019-1149-4 (PMC6534872; doi:10.1186/s12877-019-1149-4)
Supplement: Supplementary file 1 — Sample selection flow chart (DOCX 21 kb) [file 12877_2019_1149_MOESM1_ESM.docx]

Additional file 1. Sample selection flow chart
