# Supplementary material for: Body weight, frailty, and chronic pain in older adults: a cross-sectional study
Source: BMC Geriatr. 2019 May 24;19:143. doi: 10.1186/s12877-019-1149-4 (PMC6534872; doi:10.1186/s12877-019-1149-4)
Supplement: Supplementary file 2 — Adjusted association between BMI and frailty among US older adults using NHANES 2011–2016 (DOCX 20 kb) [file 12877_2019_1149_MOESM2_ESM.docx]

Additional file 2. Adjusted association between BMI and frailty among US older adults using NHANES 2011-2016 (N=3,648)

| **Risk Factors** | | **Frailty vs. Non-frailty** | | |
| --- | --- | --- | --- | --- |
|  | | **Adjusted PR** | | **95% CI** |
| **BMI** | |  | |  |
| Normal | | 1.00 | | - |
| Underweight | | 1.25 | | 1.13-1.38 |
| Overweight | | 0.94 | | 0.88-1.00 |
| Obese | | 1.09 | | 1.02-1.16 |
| **Age group, y** | |  |  |  |
| 65-69 | | 1.00 | | - |
| 70-79 | | 1.14 | | 1.07-1.22 |
| ≥ 80 | | 1.36 | | 1.27-1.46 |
| **Gender** | |  | |  |
| Male | | 1.00 | | - |
| Female | | 1.07 | | 1.02-1.12 |
| **Race/Ethnicity** | |  |  |  |
| Non-Hispanic White | | 1.00 | | - |
| Non-Hispanic Black | | 1.02 | | 0.95-1.09 |
| Mexican American | | 1.08 | | 1.00-1.18 |
| Other Hispanic | | 1.09 | | 1.00-1.18 |
| Others^a^ | | 1.02 | | 0.93-1.12 |
| **Education^b^** | |  | |  |
| < High school | | 1.00 | | - |
| High school | | 0.96 | | 0.90-1.02 |
| Some college | | 0.95 | | 0.89-1.01 |
| College | | 0.88 | | 0.81-0.95 |
| **Family income-to-poverty ratio** | | | |  |
| < 1 | | 1.00 | | - |
| 1 - ≤ 2 | | 0.94 | | 0.89-0.99 |
| >2 - < 4 | | 0.87 | | 0.81-0.93 |
| ≥ 4 | | 0.85 | | 0.78-0.92 |
| **Alcohol use^b^** | |  | |  |
| Nonuse | | 1.00 | | - |
| Moderate use | | 0.93 | | 0.87-1.00 |
| Heavy use | | 0.89 | | 0.82-0.97 |
| **Smoking^b^** | |  | |  |
| Never smoker | | 1.00 | | - |
| Former smoker | | 1.06 | | 1.00-1.12 |
| Current smoker | | 1.12 | | 1.03-1.23 |
| **Cancer^b^** |  | |  |  |
| No | | 1.00 | | - |
| Yes | | 1.26 | | 1.20-1.33 |
| **Number of chronic conditions^c^** | | | |  |
| 0 | | 1.0 | | - |
| 1 | | 2.60 | | 1.93-3.50 |
| 2 | | 4.95 | | 3.72-6.61 |
| ≥3 | | 6.93 | | 5.22-9.21 |

*Abbreviations.* BMI=Body mass index; PR=Prevalence ratio; CI=Confidence interval.

^a^ Others included Asian, the natives of North American, multi-racial, and individuals with other or unknown races and ethnicities.

^b^ Imputed those with missing values for regression analysis.

^c^ Chronic conditions of interest include arthritis, osteoporosis, hypertension, diabetes, coronary heart disease, heart attack, thyroid condition, stroke, angina, depression, heart failure, and kidney disease.
