# Supplementary material for: Body weight, frailty, and chronic pain in older adults: a cross-sectional study
Source: BMC Geriatr. 2019 May 24;19:143. doi: 10.1186/s12877-019-1149-4 (PMC6534872; doi:10.1186/s12877-019-1149-4)
Supplement: Supplementary file 3 — Adjusted association between BMI and frailty among US older adults who had no missing values on covariates of interest (DOCX 13 kb) [file 12877_2019_1149_MOESM3_ESM.docx]

Additional file 3. Adjusted^a^ association between BMI and frailty among US older adults who had no missing values on covariates of interest (N=2,333)

| **BMI** | **Frailty vs. Non-frailty** | |
| --- | --- | --- |
|  | **Adjusted PR** | **95% CI** |
| Normal | 1.00 | - |
| Underweight | 1.35 | 1.21-1.51 |
| Overweight | 1.03 | 0.94-1.12 |
| Obese | 1.24 | 1.14-1.36 |

*Abbreviations.* Abbreviations: BMI=Body mass index; PR=Prevalence ratio; CI=Confidence interval.

^a^ Adjusted for age, gender, race/ethnicity, education level, family income-to-poverty ratio, alcohol use, smoking, cancer, and number of chronic conditions.
