# Supplementary material for: Body weight, frailty, and chronic pain in older adults: a cross-sectional study
Source: BMC Geriatr. 2019 May 24;19:143. doi: 10.1186/s12877-019-1149-4 (PMC6534872; doi:10.1186/s12877-019-1149-4)
Supplement: Supplementary file 4 — Adjusted association between BMI and chronic pain, with and without adjustment of frailty among older adults who had no missing values on covariates of interest (DOCX 15 kb) [file 12877_2019_1149_MOESM4_ESM.docx]

Additional file 4. Adjusted^a^ association between BMI and chronic pain, with and without adjustment of frailty among older adults who had no missing values on covariates of interest (N=2,333)

| **BMI** | **Chronic Pain** | | | | | **% Decrease of PR^b^** |
| --- | --- | --- | --- | --- | --- | --- |
|  | **Model 1 without Frailty** | |  | **Model 2 with Frailty** | |  |
|  | **PR** | **95% CI** |  | **PR** | **95% CI** |  |
| Normal | 1.00 | - |  | 1.00 | - |  |
| Underweight | 1.75 | 1.15-2.66 |  | 1.52 | 1.00-2.30 | 13.3% |
| Overweight | 1.17 | 0.87-1.56 |  | 1.15 | 0.86-1.53 | 1.6% |
| Obese | 1.42 | 1.05-1.92 |  | 1.30 | 0.97-1.76 | 8.3% |

*Abbreviations.* BMI=Body mass index; PR=Prevalence rate ratio; CI=Confidence interval.

^a^ Adjusted for age, gender, race/ethnicity, education level, family income-to-poverty ratio, alcohol use, smoking, cancer, and number of chronic conditions.

^b^ Calculated as [(PR from model 1- PR from model 2) divided by PR from model 1] multiplied by 100%.
