# Supplementary material for: Body weight, frailty, and chronic pain in older adults: a cross-sectional study
Source: BMC Geriatr. 2019 May 24;19:143. doi: 10.1186/s12877-019-1149-4 (PMC6534872; doi:10.1186/s12877-019-1149-4)
Supplement: Supplementary file 5 — Adjusted association between BMI and chronic pain in older adults who had no missing values on covariates of interest, overall and stratified by frailty (DOCX 14 kb) [file 12877_2019_1149_MOESM5_ESM.docx]

Additional file 5. Adjusted^a^ association between BMI and chronic pain in older adults who had no missing values on covariates of interest, overall and stratified by frailty

| **BMI** | **Overall**  **(N=2,333)** | | **Frailty**  **(n=1,252)** | | **Non-Frailty**  **(n=1,081)** | |
| --- | --- | --- | --- | --- | --- | --- |
|  | **Chronic Pain** | | | | | |
|  | **PR** | **95% CI** | **PR** | **95% CI** | **PR** | **95% CI** |
| Normal | 1.00 | | 1.00 | | 1.00 | |
| Underweight | 1.52 | 1.00-2.30 | 1.59 | 1.02-2.48 | 1.86 | 0.45-7.62 |
| Overweight | 1.15 | 0.86-1.53 | 1.26 | 0.91-1.74 | 0.84 | 0.44-1.59 |
| Obese | 1.30 | 0.97-1.76 | 1.38 | 0.98-1.93 | 1.24 | 0.64-2.40 |
| Overall P for the interaction term (BMI* frailty) =0.946 | | | | | | |

*Abbreviations.* BMI=Body mass index; PR=Prevalence ratio; CI=Confidence interval.

^a^ Adjusted for age, gender, race/ethnicity, education level, family income-to-poverty ratio, alcohol use, smoking, cancer, and number of chronic conditions.
