# Supplementary material for: Body weight, frailty, and chronic pain in older adults: a cross-sectional study
Source: BMC Geriatr. 2019 May 24;19:143. doi: 10.1186/s12877-019-1149-4 (PMC6534872; doi:10.1186/s12877-019-1149-4)
Supplement: Supplementary file 6 — Comparison of characteristics of older respondents in 1999–2004 NHANES versus in 2011–2016 NHANES (DOCX 21 kb) [file 12877_2019_1149_MOESM6_ESM.docx]

Additional file 6. Comparison of characteristics of older respondents in 1999-2004 NHANES versus in 2011-2016 NHANES

| **Characteristics** | **1999-2004**  **(%)** | **2011-2016** | **P-**  **Value** |
| --- | --- | --- | --- |
|  |  | **(%)** |  |
|  | **N=3,704^a^** | **N=3,648^a^** |  |
| **BMI** |  |  | <.0001 |
| Normal | 27.6 | 24.8 |  |
| Underweight | 7.2 | 3.2 |  |
| Overweight | 37.6 | 36.2 |  |
| Obese | 27.6 | 35.7 |  |
| **Age group, y** |  |  | <.0001 |
| 65-69 | 29.1 | 34.2 |  |
| 70-79 | 47.0 | 42.0 |  |
| ≥ 80 | 23.8 | 23.8 |  |
| **Male sex** | 42.7 | 44.1 | 0.334 |
| **Race/Ethnicity** |  |  | <.0001 |
| Non-Hispanic White | 82.8 | 78.1 |  |
| Non-Hispanic Black | 7.7 | 8.0 |  |
| Mexican American | 2.8 | 3.7 |  |
| Other Hispanic | 4.1 | 3.5 |  |
| Others^b^ | 2.6 | 6.6 |  |
| **Education** |  |  | <.0001 |
| < High school | 32.0 | 19.4 |  |
| High school | 29.3 | 22.2 |  |
| Some college | 22.2 | 30.5 |  |
| College | 16.5 | 27.9 |  |
| **Family income-to-poverty ratio** |  |  | 0.001 |
| < 1 | 21.3 | 19.8 |  |
| 1 - ≤ 2 | 28.4 | 23.2 |  |
| >2 - <4 | 29.6 | 27.0 |  |
| ≥ 4 | 20.8 | 29.9 |  |
| **Alcohol use** |  |  | 0.014 |
| No use | 30.9 | 20.8 |  |
| Moderate use | 54.5 | 60.9 |  |
| Heavy use | 14.7 | 18.2 |  |
| **Smoking** |  |  | 0.120 |
| Never smoker | 49.2 | 49.5 |  |
| Former smoker | 41.8 | 41.7 |  |
| Current smoker | 9.0 | 8.8 |  |
| **Cancer (Yes)** | 23.6 | 28.7 | 0.000 |
| **Number of chronic conditions** |  |  | <.0001 |
| 0 | 15.3 | 12.5 |  |
| 1 | 28.2 | 26.3 |  |
| 2 | 25.9 | 28.4 |  |
| ≥3 | 30.5 | 32.8 |  |
| **Frailty (Yes)** | 57.2 | 55.4 | 0.283 |

*Abbreviations.* BMI=Body mass index.

^a^ The similar inclusion criteria were used for both study sample: 1) ≥ 65 years old, 2) non-zero sample weight, 3) have complete information on BMI and frailty index.

^b^ Others included Asian, the natives of North American, multi-racial, and individuals with other or unknown races and ethnicities
